# PEGylation Potentiates the Effectiveness of an Antagonistic Peptide That Targets the EphB4 Receptor with Nanomolar Affinity

**DOI:** 10.1371/journal.pone.0028611

**Published:** 2011-12-14

**Authors:** Roberta Noberini, Sayantan Mitra, Ombretta Salvucci, Fatima Valencia, Srinivas Duggineni, Natalie Prigozhina, Ke Wei, Giovanna Tosato, Ziwei Huang, Elena B. Pasquale

**Affiliations:** 1 Sanford-Burnham Medical Research Institute, La Jolla, California, United States of America; 2 Laboratory of Cellular Oncology, Center for Cancer Research, National Cancer Institute, National Institutes of Health, Bethesda, Maryland, United States of America; 3 Department of Pharmacology, State University of New York Upstate Cancer Research Institute, State University of New York, Syracuse, New York, United States of America; 4 Biology Department, University of San Diego, San Diego, California, United States of America; 5 Department of Pathology, University of California San Diego, San Diego, California, United States of America; University of Helsinki, Finland

## Abstract

The EphB4 receptor tyrosine kinase together with its preferred ligand, ephrin-B2, regulates a variety of physiological and pathological processes, including tumor progression, pathological forms of angiogenesis, cardiomyocyte differentiation and bone remodeling. We previously reported the identification of TNYL-RAW, a 15 amino acid-long peptide that binds to the ephrin-binding pocked of EphB4 with low nanomolar affinity and inhibits ephrin-B2 binding. Although ephrin-B2 interacts promiscuously with all the EphB receptors, the TNYL-RAW peptide is remarkably selective and only binds to EphB4. Therefore, this peptide is a useful tool for studying the biological functions of EphB4 and for imaging EphB4-expressing tumors. Furthermore, TNYL-RAW could be useful for treating pathologies involving EphB4-ephrin-B2 interaction. However, the peptide has a very short half-life in cell culture and in the mouse blood circulation due to proteolytic degradation and clearance by the kidneys and reticuloendothelial system. To overcome these limitations, we have modified TNYL-RAW by fusion with the Fc portion of human IgG1, complexation with streptavidin or covalent coupling to a 40 KDa branched polyethylene glycol (PEG) polymer. These modified forms of TNYL-RAW all have greatly increased stability in cell culture, while retaining high binding affinity for EphB4. Furthermore, PEGylation most effectively increases peptide half-life *in vivo*. Consistent with increased stability, submicromolar concentrations of PEGylated TNYL-RAW effectively impair EphB4 activation by ephrin-B2 in cultured B16 melanoma cells as well as capillary-like tube formation and capillary sprouting in co-cultures of endothelial and epicardial mesothelial cells. Therefore, PEGylated TNYL-RAW may be useful for inhibiting pathological forms of angiogenesis through a novel mechanism involving disruption of EphB4-ephrin-B2 interactions between endothelial cells and supporting perivascular mesenchymal cells. Furthermore, the PEGylated peptide is suitable for other cell culture and *in vivo* applications requiring prolonged EphB4 receptor targeting.

## Introduction

EphB4 is a member of the large Eph receptor tyrosine kinase family. Eph receptors have been implicated in a wide variety of physiological and pathological processes, and are therefore considered promising drug target candidates [1]. The EphB4 receptor preferentially binds the transmembrane ligand, ephrin-B2, at sites of cell-cell contact. The interaction initiates bidirectional cellular responses through both EphB4 (“forward” signals) and ephrin-B2 (“reverse” signals).

EphB4 is highly expressed in most cancer cell types, including prostate, breast, ovarian, colorectal, lung and bladder cancers, melanoma and mesothelioma [2], [3], [4], [5], [6], [7], [8]. EphB4-ephrin-B2 bidirectional signaling appears to promote the malignancy of certain cancers, such as melanoma [9], [10], [11]. However, the role of the EphB4/ephrin-B2 system in other cancer cell types is controversial [1], [2], [4], [7], [12].

EphB4-ephrin-B2 interaction is also known to play a critical role in angiogenesis, including blood vessel remodeling during embryonic development, tumor vascularization and other forms of pathological angiogenesis [2], [13], [14]. Ephrin-B2 is widely expressed in tumor blood vessels, where it is upregulated by hypoxia and vascular endothelial growth factor (VEGF). Ephrin-B2 reverse signaling, triggered by contact with EphB4 and other EphB receptors, plays an important role in angiogenic responses. For example, it promotes the migration/invasion and proliferation/survival of cultured endothelial cells [15], [16], [17]. In addition, recent studies have shown that ephrin-B2 reverse signaling is required for vascular endothelial growth factor (VEGF) receptor internalization in endothelial cells, which is critical for VEGF-dependent angiogenesis [18], [19]. Ephrin-B2 reverse signaling also plays an important role in perivascular mesenchymal cells, including pericytes and vascular smooth muscle cells, and regulates their association with endothelial cells *in vivo* leading to vessel maturation and stabilization [20], [21]. Thus, an important role of EphB4 during tumor progression is to promote ephrin-B2 reverse signaling in the vasculature. This role may be fulfilled by EphB4 present in tumor cells or co-expressed in vascular cells [13], [14], [15], [22]. Additionally, ephrin-B2-induced EphB4 forward signaling in endothelial cells likely contributes to tumor angiogenesis [13], [14], [16], [19], [23], [24].

The interaction between EphB4 expressed in circulating tumor cells and ephrin-B2 expressed in endothelial cells has also been reported to mediate site-specific metastatic dissemination [8]. Other roles of EphB4-ephrin-B2 bidirectional signaling include regulation of bone remodeling [25] and cardiomyocyte differentiation [26], [27].

Inhibiting EphB4-ephrin-B2 interaction could therefore be useful for diverse medical applications. For example, administration of soluble monomeric EphB4 extracellular domain, which interferes with the binding of ephrin-B2 to EphB receptors and inhibits bidirectional signaling, was shown to inhibit tumor growth and tumor angiogenesis in several mouse tumor xenograft models as well as neovascularization in a model of retinopathy [28], [29], [30], [31], [32]. A 15 amino acid-long EphB4 antagonistic peptide, TNYL-RAW (TNYLFSPNGPIARAW), could represent an alternative to the large EphB4 extracellular domain. TNYL-RAW is a modified version of a 12 amino acid-long peptide identified by phage display [33]. It binds to EphB4 with remarkably high affinity (K_D_ of 3 nM for mouse EphB4 as measured by surface plasmon resonance [34] and 70 nM for human EphB4 as measured by isothermal titration calorimetry [35]). TNYL-RAW binds in the hydrophobic pocket that represents the high affinity binding site for ephrin-B2 and selectively binds to EphB4 but not any other Eph receptor [33], [35].

The TNYL-RAW peptide has been shown to inhibit EphB4 tyrosine phosphorylation (activation) induced by ephrin-B2 in cultured MCF7 breast cancer cells [33] and human umbilical vein endothelial cells (HUVECs) [36]. Furthermore, it inhibits ephrin-B2-induced retraction of HUVECs and capillary-like tube formation on Matrigel [21], [36], [37]. Hence, this peptide could be useful to inhibit EphB4 pathological activities.

Here we show that, like many other peptides, TNYL-RAW is rapidly lost from cell culture medium and when administered *in vivo*. This limits its possible research, therapeutic and diagnostic applications. Therefore, we have investigated approaches to increase its half-life in cell culture and *in vivo*. These studies identify PEGylated TNYL-RAW as an improved form of the peptide that retains high binding affinity for EphB4 but has greatly decreased susceptibility to proteolytic degradation and increased permanence in the mouse blood circulation. Importantly, PEGylation is a modification compatible with the use of TNYL-RAW for preclinical as well as clinical studies.

## Results

### The TNYL-RAW peptide has a short half-life in cell culture medium and in the mouse circulation

We previously reported that the TNYL-RAW peptide inhibits EphB4-ephrin-B2 binding with an IC_50_ value of ∼15 nM in ELISA assays [33]. However, much higher peptide concentrations (10–100 µM) are needed to substantially inhibit ephrin-B2-induced EphB4 tyrosine phosphorylation and downstream effects in cultured cells [21], [26], [33], [36], [37]. An explanation for the discrepancy in the potency of the peptide in biochemical and cell culture assays could be that TNYL-RAW is susceptible to degradation by proteases present in cell culture medium. For example, trypsin-like proteases are likely to cleave the peptide between R_13_ and A_14_ [38]. The consequent loss of W_15_, which is involved in multiple interactions with the ephrin-binding pocket of EphB4 [35], would be expected to result in a dramatic decrease in affinity.

**Figure 1:**
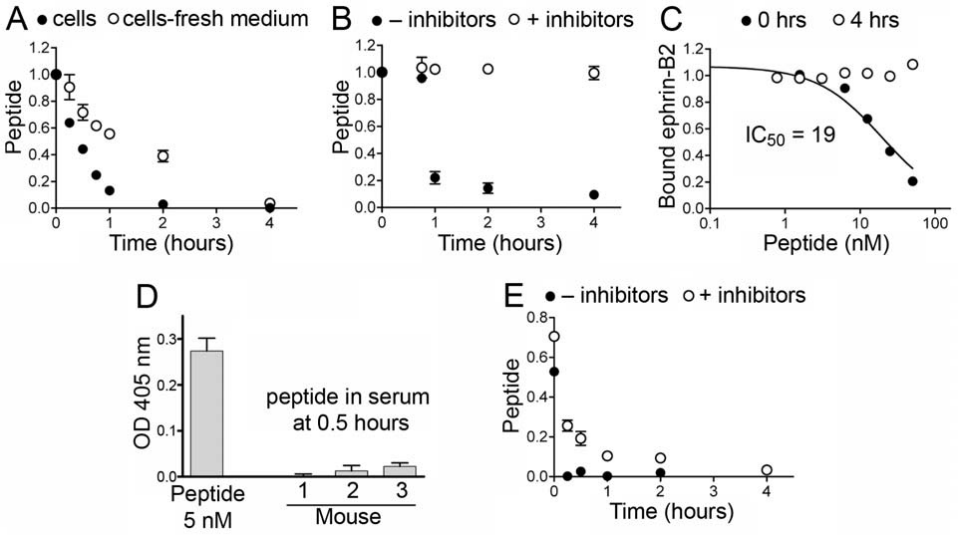
The TNYL-RAW peptide is rapidly lost in cell culture medium and from the mouse circulation. (A) Biotinylated TNYL-RAW peptide was incubated with cultured PC3 prostate cancer cells grown in the same medium for 3 days or in the culture medium freshly replaced just before adding the peptide. Functional (EphB4- and streptavidin-binding) peptide remaining at the indicated times was captured in ELISA plates coated with EphB4 Fc and detected with streptavidin-HRP. (B) TNYL-RAW was incubated at 37°C in PC3 cell conditioned medium (without cells) with and without a mixture of protease inhibitors including aprotinin, leupeptin, pepstatin and PMSF, and detected as in (A). (C) TNYL-RAW was incubated with PC3 cell conditioned medium for 4 hours and added together with ephrin-B2 AP to ELISA wells pre-coated with EphB4 Fc. TNYL-RAW mixed with conditioned medium right before the ELISA assay (0 hrs) was used as a control. The graph shows the ratio of ephrin-B2 AP bound in the presence and in the absence of peptide. (D) Serum from 3 mice injected intravenously with 6 nmoles biotinylated TNYL-RAW was collected 30 min after peptide administration and incubated at a dilution of 1∶20 in ELISA wells pre-coated with EphB4 Fc. Based on the amount of injected TNYL-RAW and an estimated mouse serum volume of 2.5 ml, the peptide concentration in the wells would be 120 nM. TNYL-RAW at a concentration of 5 nM in similarly diluted mouse serum was used for comparison. Bound peptide was detected with streptavidin-HRP. (E) TNYL-RAW was incubated in undiluted mouse serum *ex vivo* for the indicated times and detected as described in (A). Averages from 3 measurements ± SE are shown in all the panels.

To assess the half-life of TNYL-RAW in cell culture, a biotinylated form of the peptide was incubated with PC3 prostate cancer cells that had been grown in the same medium for several days. The amount of intact TNYL-RAW remaining after different incubation times was measured in ELISA assays by capturing the peptide with immobilized EphB4 Fc and detecting it with streptavidin conjugated to horseradish peroxidase (HRP). TNYL-RAW was rapidly lost in PC3 cell culture medium, with half-lives varying from less than an hour to several hours depending on cell density and how long the cells had been grown in the culture medium (Figure 1A and data not shown). Moreover, when incubated with cells in fresh culture medium, TNYL-RAW had a longer half-life (Figure 1A), suggesting that its loss is due to degradation by proteases produced by the cells and accumulating in the culture medium rather than uptake by the cells or other mechanisms. Supporting the idea of proteolytic degradation, TNYL-RAW loss was also observed in conditioned medium without the cells (Figure 1B) and was blocked by a mixture of protease inhibitors (Figure 1B). Incubation in PC3 cell conditioned medium for several hours also abolished the ability of TNYL-RAW to inhibit ephrin-B2-EphB4 interaction in ELISA assays (Figure 1C), confirming that the peptide itself is cleaved rather than just losing the biotin tag used for detection. Rapid TNYL-RAW loss was also observed in all other cell types examined, including B16 melanoma cells, human umbilical vein endothelial cells (HUVECs) and epicardial mesothelial cells (EMCs) (Figure S1) as well as C6 glioma cells and BPH1 prostate epithelial cells (data not shown). TNYL-RAW is also very rapidly lost from the mouse circulation and in our ELISA assay it could not be detected in serum as early as 30 min after intravenous administration (Figure 1D). This may be at least in part due to proteolytic degradation because TNYL-RAW is also rapidly lost when incubated *ex vivo* at 37°C in mouse serum, an effect that can be partially counteracted by a mixture of protease inhibitors (Fig. 1E). Some peptide loss also occurs at room temperature during the ELISA assay, since less peptide is detected at the 0 time point in the absence of protease inhibitors.

### Modified forms of TNYL-RAW retain high EphB4 binding affinity

**Figure 2:**
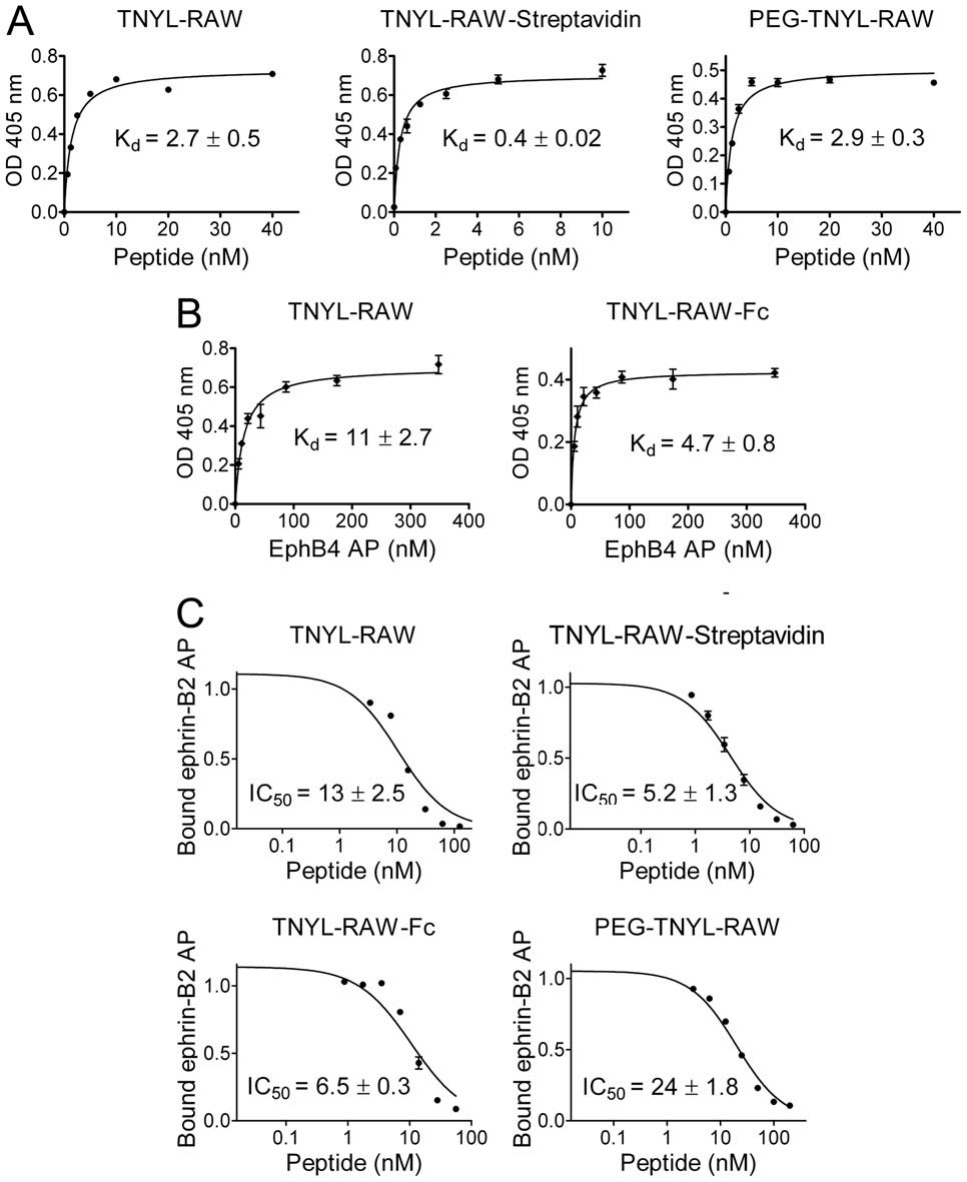
Modified forms of TNYL-RAW retain high EphB4 binding affinity and potency for inhibition of EphB4-ephrin-B2 binding. (A) Biotinylated, streptavidin-bound and PEGylated TNYL-RAW were incubated at the indicated concentrations in EphB4-coated ELISA wells. Biotinylated TNYL-RAW was detected with streptavidin-HRP, TNYL-RAW-streptavidin was detected with and anti-streptavidin antibody coupled to HRP, and PEG-TNYL-RAW was detected with an anti-PEG antibody followed by a secondary antibody conjugated to HRP. (B) The indicated concentrations of EphB4 AP were incubated in ELISA wells pre-coated with streptavidin and biotinylated TNYL-RAW (left) or an anti-IgG antibody and TNYL-RAW-Fc (right). K_d_ values are based on EphB4 AP concentrations calculated from AP activity. (C) The different forms of TNYL-RAW were incubated at the indicated concentrations together with a constant amount of ephrin-B2 AP in ELISA wells pre-coated with EphB4 Fc. The ratio of ephrin-B2 AP bound in the presence and in the absence of peptide is shown. The graphs show averages ± SE from triplicate measurements in representative experiments, while the K_d_ and IC_50_ values are calculated from 3 to 11 experiments.

We investigated several modifications that might improve the resistance of the TNYL-RAW peptide to proteolytic degradation and increase its half-life in the blood circulation. We generated TNYL-RAW fused to the Fc portion of human IgG_1_ by purification from the culture medium of transiently transfected 293 human embryonal kidney (HEK) cells, because Fc fusion proteins typically have increased resistance to proteolysis and a long half-life *in vivo* [39], [40]. We also modified the biotinylated synthetic TNYL-RAW by complexation with streptavidin or by covalent coupling to a 40 KDa branched PEG polymer. Streptavidin is a bacterial tetrameric protein of approximately 50 KDa that binds biotin with very high affinity and has also been used to stabilize peptides *in vivo* [41], [42]. Attachment to large PEG molecules is a modification often used in the pharmacological industry to decrease proteolytic degradation and *in vivo* clearance of peptides, proteins and small molecules [40], [43]. To minimize possible negative effects of the large branched PEG molecule on the ability of TNYL-RAW to bind EphB4, we attached it to the N terminus of TNYL-RAW, because the N-terminal T_1_ and N_2_ amino acids are not involved in the interaction with EphB4 [35]. By measuring TNYL-RAW binding to immobilized EphB4 or EphB4 binding to immobilized TNYL-RAW in ELISA assays, we confirmed that the binding affinity of all three modified forms of the peptide is not substantially affected compared to the unmodified biotinylated peptide (Figure 2A,B). The apparent increase in affinity observed for TNYL-RAW bound to streptavidin or fused to Fc is likely due to the multimeric nature of these molecules, because TNYL-RAW-Fc is a dimer and TNYL-RAW-streptavidin is a tetramer. ELISA competition experiments further confirmed that none of the modifications substantially affects the ability of TNYL-RAW to inhibit EphB4-ephrin-B2 binding (Figure 2C). Therefore, the TNYL-RAW peptide fused to Fc, bound to streptavidin or covalently coupled to 40 KDa PEG remains a potent EphB4 antagonist.

### Modified forms of TNYL-RAW have increased half-life in cell culture medium and mouse blood

**Figure 3:**
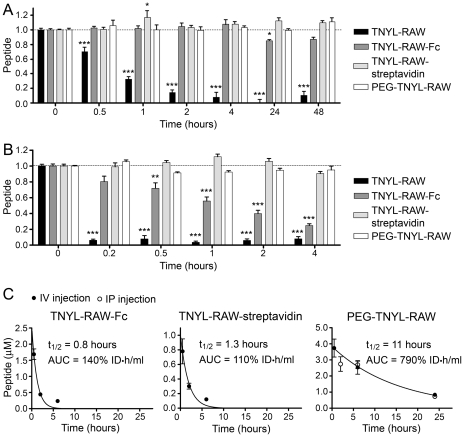
Modified forms of TNYL-RAW have increased stability in cell culture medium and in the mouse circulation. (A, B) Biotinylated, streptavidin-bound, fused to Fc and PEGylated TNYL-RAW were incubated in medium conditioned by PC3 prostate cancer cells (A) or mouse serum (B). Functional peptide remaining at the indicated times was captured in ELISA plates and quantified. Biotinylated TNYL-RAW was captured on ELISA wells pre-coated with EphB4 Fc and detected with Streptavidin-HRP. TNYL-RAW-streptavidin was captured on wells pre-coated with EphB4 Fc and detected with an anti-streptavidin antibody coupled to HRP. TNYL-RAW-Fc was captured on wells coated with an anti-Fc antibody and detected with EphB4 AP. PEG-TNYL-RAW was captured on wells coated with EphB4 Fc and detected with anti-PEG antibody followed by a secondary antibody conjugated to HRP. Normalized averages from 6–9 measurements ± SE are shown. Peptide amounts at different time points were compared to those at time 0 by one-way ANOVA and Dunnett's post test. *P<0.05, **P<0.001 and ***P<0.001. (C) Blood from mice injected intravenously (IV) or intraperitoneally (IP) with 2.1 nmoles TNYL-RAW-Fc, 1.5 nmoles TNYL-RAW-streptavidin or 6 nmoles of PEG-TNYL-RAW was collected and peptide levels in the serum were measured in ELISA assays as described in (A). Peptide concentrations in serum were calculated based on a standard curve generated using serum containing known TNYL-RAW concentrations. Areas under the curve (AUC) values were calculated as described in the Materials and Methods. Averages from blood collected from 3 mice ± SE are shown.

Fusion to Fc, complexation with streptavidin and PEGylation all dramatically improve the half-life of TNYL-RAW in cell culture medium, as indicated by the absence of peptide degradation after incubation in PC3 cell conditioned medium for up to 48 hours (Figure 3A) and much decreased degradation after incubation in mouse serum (Figure 3B). Of note, TNYL-RAW-Fc was not as stable in mouse serum as the streptavidin-bound or PEGylated peptide. The three modifications also increased the persistence of TNYL-RAW in the mouse blood circulation after intravenous injection. While TNYL-RAW was undetectable in serum prepared from blood collected 30 min after peptide injection (Figure 1D), the circulation half-life of TNYL-RAW-Fc and TNYL-RAW-streptavidin was ∼1 hour (area under the curve (AUC) = 140% and 110% of the injected dose•hours/ml, respectively) and the half-life of intravenously injected PEG-TNYL-RAW was ∼11 hours (AUC = 790% of the injected dose•hours/ml) (Figure 3C). We also found that even though as expected PEG-TNYL-RAW injected intraperitoneally enters the circulation more slowly than the peptide injected intravenously, it nevertheless reaches similar blood levels after 6 and 24 hours (Figure 3C, right panel). Thus, intraperitoneal injection can be used as a more convenient route of administration for *in vivo* studies.

### PEGylated TNYL-RAW inhibits EphB4 and ephrin-B2 phosphorylation in cells

**Figure 4:**
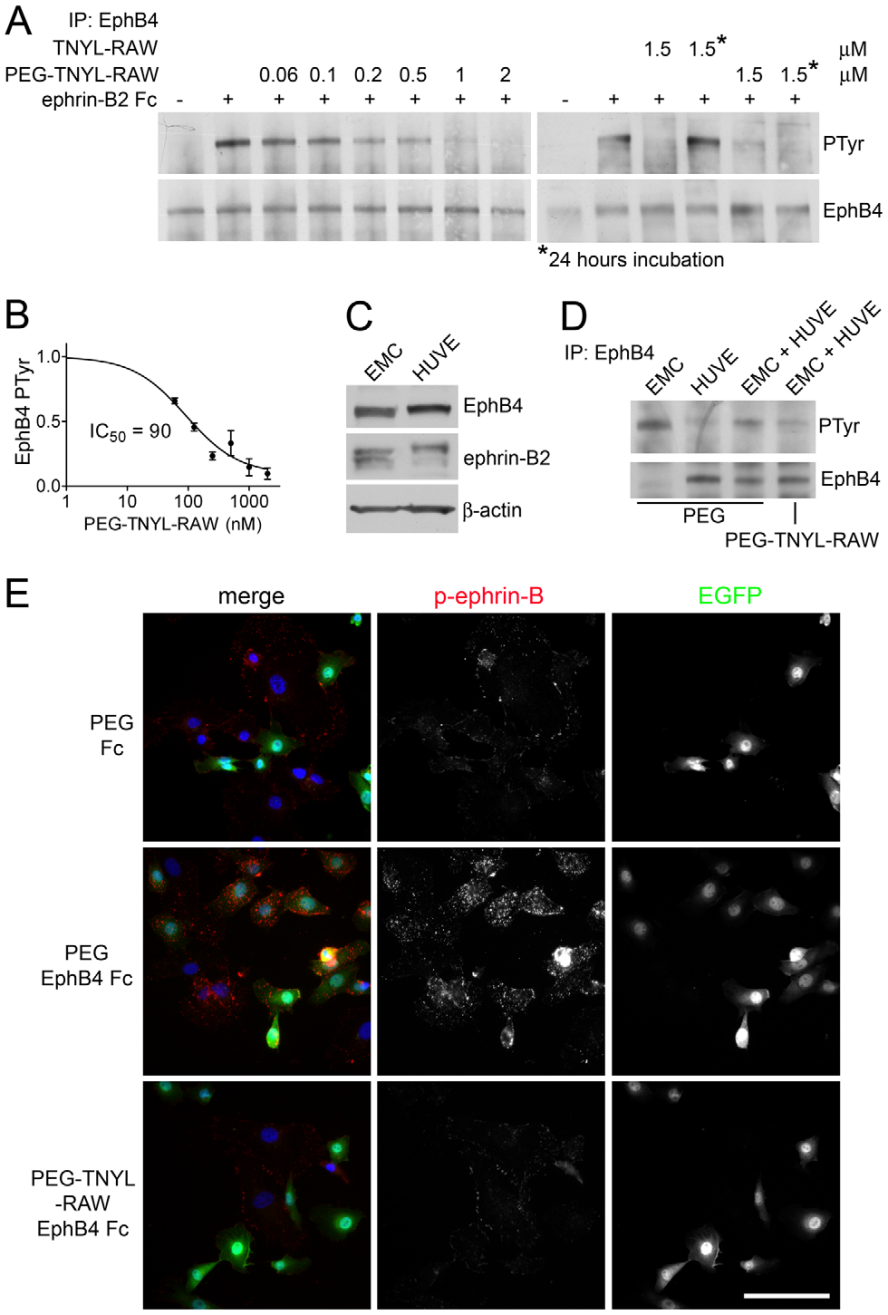
PEGylated TNYL-RAW inhibits tyrosine phosphorylation of EphB4 and ephrin-B2. (A) B16 melanoma cells pretreated with the indicated concentrations of PEG-TNYL-RAW or TNYL-RAW for 15 min or 24 hours were stimulated with 1.5 µg/ml preclustered ephrin-B2 Fc (+) or Fc as a control (−) for 20 min in the continued presence of the peptide. EphB4 immunoprecipitates were probed with anti-phosphotyrosine antibody (PTyr) and reprobed for EphB4. (B) The inhibition curve shows the relative levels of EphB4 phosphorylation in the presence of different concentrations of PEG-TNYL-RAW, which were quantified from immunoblots and normalized to the amount of immunoprecipitated EphB4. Error bars represent the standard error from 3–6 experiments. (C) HUVEC and EMC lysates were probed for EphB4, ephrin-B2 (band at ∼45 Kd detected with a pan-ephrin-B antibody) and ß-actin as a loading control. It is not known why the ephrin-B2 band appears as a more prominent doublet in EMCs than HUVECs. (D) HUVECs and EMCs were cultured individually or mixed at a 1∶1 ratio in the presence of 1.5 µM PEG-TNYL-RAW or PEG control. EphB4 immunoprecipitates were probed with an anti-phosphotyrosine antibody (PTyr) and reprobed for EphB4. (E) HUVECs and EMCs, which express EGFP, were cultured at a 1∶1 ratio for 15 hours in the presence of 1.5 µM PEG-TNYL-RAW or PEG control. The cells were then stimulated with 1.5 µg/ml preclustered EphB4 Fc or Fc as a control for 20 min in the continued presence of the peptide or PEG. The cells were stained for phospho-ephrin-B (red), which likely corresponds to the phosphorylated form of the EphB4 preferred ligand ephrin-B2, and nuclei were labeled with DAPI (blue). Scale bar = 50 µM. Fluorescence intensity from 6 micrographs per condition was quantified. The values obtained (expressed in arbitrary units ± standard error) are: PEG & Fc, 24±2.6; PEG & EphB4 Fc, 44±3.6; PEG-TNYL-RAW & EphB4 Fc, 20±2.5. The fluorescence of cells treated with PEG-TNYL-RAW & EphB4 Fc was significantly (P<0.001) different from that of cells treated with PEG & EphB4 Fc, but not from that of cells treated with PEG & Fc, by one-way ANOVA and Bonferroni's post test.

Consistent with its greatly improved half-life in cell culture medium, PEG-TNYL-RAW inhibits ephrin-B2-induced tyrosine phosphorylation of endogenous EphB4 expressed in B16 melanoma cells with an IC_50_ value of 90 nM, and 2 µM peptide are sufficient to reduce receptor phosphorylation to undetectable levels (Figure 4A, B). Furthermore, PEG-TNYL-RAW but not the unmodified peptide, retains its inhibitory activity even after 24 hours of incubation with B16 cells (Figure 4A). Prolonged incubation with PEG-TNYL-RAW also inhibits endogenous EphB4 phosphorylation in mixed cultures of epicardial mesothelial cells (EMCs) and human umbilical vein endothelial cells (HUVECs), two cell types that express both EphB4 and ephrin-B2 (Figure 4C, D) and can assemble together into capillary-like structures when cultured together on Matrigel [44], [45], [46] (see below). Interestingly, the peptide also inhibits tyrosine phosphorylation of ephrin-B2 induced by treatment of EMCs and HUVECs with EphB4 Fc, a soluble dimeric form of the EphB4 extracellular domain fused to Fc that should preferentially activate ephrin-B2 among the ephrin-B ligands (Figure 4E). Ephrin-B2 phosphorylation was measured by immunofluorescence microscopy using a phospho-ephrin-B antibody, which performed better in this type of assay than in immunoblotting experiments. Thus, PEG-TNYL-RAW can be used to inhibit both forward and reverse signals generated by the EphB4-ephrin-B2 complex.

### Low micromolar concentrations of PEGylated TNYL-RAW inhibit capillary-like tube formation and capillary sprouting

**Figure 5:**
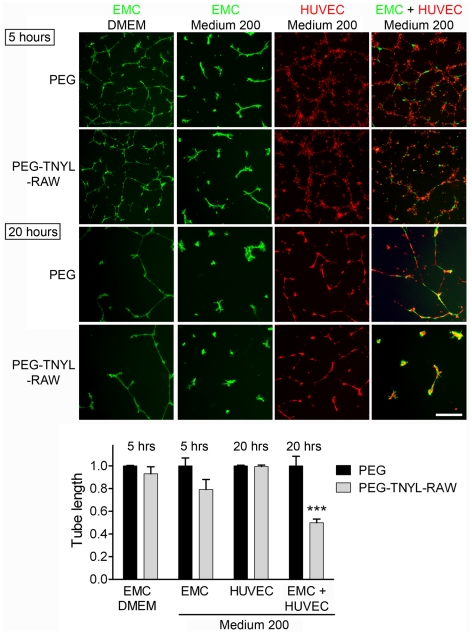
PEGylated TNYL-RAW inhibits capillary-like tube formation in co-cultured HUVECs and EMCs. EMCs expressing EGFP and cultured in DMEM or complete Medium 200 and HUVECs labeled with CellTracker™ Orange were plated on Matrigel individually or mixed at a 1∶2 ratio and imaged 5 and 20 hours later. The effect of 5 µM PEG-TNYL-RAW or an equal amount of PEG control on tube formation was analyzed. The histogram shows average tube lengths quantified from 2–3 micrographs at 5 hours for EMCs and 20 hours for HUVECs and HUVEC + EMC co-cultures and normalized to the average for the PEG control. Error bars represent the standard error from tube length measured from 3–4 wells. Tube length in the presence of PEG-TNYL-RAW was compared to that in the presence of PEG by one-way ANOVA and Bonferroni's post test. ***P<0.001. PEG-TNYL-RAW showed a trend towards decreasing tube formation in EMCs grown in Medium 200, which however did not reach significance (P = 0.11 by t-test). Scale bar = 250 µM.

**Figure 6:**
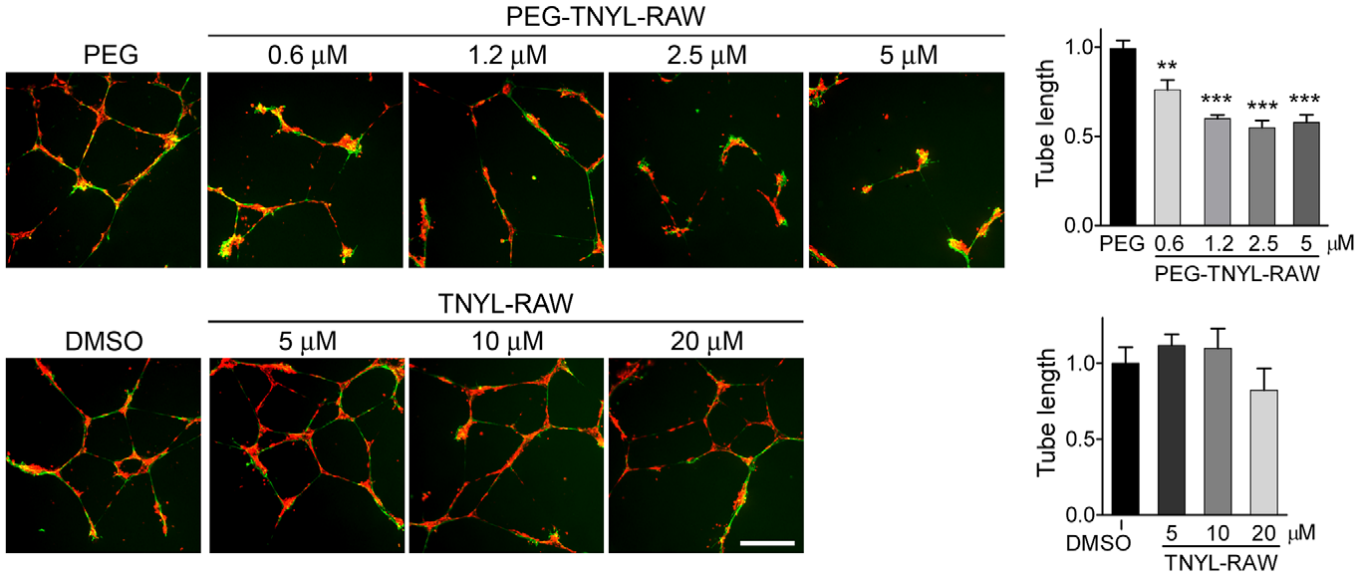
PEGylation increases the effectiveness of the TNYL-RAW peptide in inhibiting capillary-like tube formation by co-coltured HUVECs and EMCs. HUVECs labeled with CellTracker™ Orange and EMCs expressing EGFP were plated on Matrigel in complete Medium 200 at a 2∶1 ratio and imaged 15 hours later. The effect of different concentrations of PEG-TNYL-RAW or TNYL-RAW on tube formation was analyzed. The histograms show the average tube lengths for the different peptide treatments normalized to the average for the PEG or DMSO controls. Error bars represent the standard error from 3–4 wells. Tube lengths for PEG-TNYL-RAW or TNYL-RAW were compared to the PEG or DMSO control by one-way ANOVA and Dunnett's post test. **P<0.001; ***P<0.001. TNYL-RAW at 20 µM showed a trend towards decreasing tube formation, which however did not reach significance (P = 0.40 by t-test). Scale bar = 250 µM.

**Figure 7:**
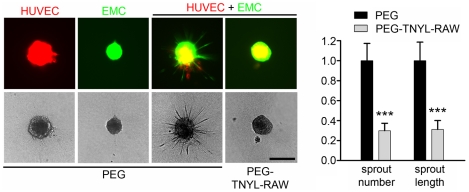
PEGylated TNYL-RAW inhibits capillary sprouting in co-coltured HUVECs and EMCs. Collagen embedded spheroids generated with HUVECs expressing mCherry, EMCs expressing EGFP or a 1∶1 mixture of the two cell types were treated with 5 µM PEG-TNYL-RAW or PEG for 2 days. The number of sprouts and the cumulative sprout length in the HUVE + EMC spheroids were normalized to the average for the PEG control. The histogram shows averages from 40–45 spheroids ± SE. The values obtained for spheroids treated with PEG-TNYL-RAW were compared to those with PEG control by one-way ANOVA. ***P<0.001. Scale bar = 250 µM.

Ephrin-B2 reverse signaling has been shown to play an important role in the interaction between endothelial cells and vascular mural cells such as pericytes and smooth muscle cells [20], [21], [30], [31]. EMCs express smooth muscle actin (Figure S2) and after 20 hours on Matrigel form capillary-like structures together with HUVECs, as expected for vascular support cells (Figure 5). We found that PEG-TNYL-RAW at a concentration of 5 µM disrupts the tubular organization of the mixed endothelial-mural cell structures (Figure 5), whereas the PEG control had no effect compared to no treatment (data not shown). PEG-TNYL-RAW is also effective at submicromolar concentrations, whereas unmodified TNYL-RAW does not have significant effects at the concentrations tested (up to 20 µM; Figure 6). Interestingly, although both HUVECs and EMCs can also form capillary-like tubes on Matrigel when cultured individually, TNYL-RAW did not significantly affect the integrity of these structures (Figure 5), which excludes non-specific toxic effects of the peptide on the cells. Notably, EMCs form tubes more rapidly than HUVECs, but their tubes spontaneously disintegrate by 15–20 hours in the absence of co-cultured HUVECs. PEG-TNYL-RAW also reduced the capillary sprouts formed over a period of two days by spheroids containing both HUVECs and EMCs and embedded in a collagen gel (Figure 7). These effects imply that EphB4 is a particularly important partner for ephrin-B2 in the interplay between endothelial and mural cells and suggest that the TNYL-RAW peptide can be used to effectively inhibit angiogenic responses involving the two cell types.

## Discussion

Peptides are known to have a short half-life in cell culture or when systemically administered *in vivo*, mainly due to proteolytic degradation and clearance by the kidneys and reticuloendothelial system [40], [43], [47], [48]. We indeed found that the EphB4-targeting peptide, TNYL-RAW, is rapidly lost in cell culture medium and in the mouse blood circulation. This is in agreement with the blood pharmacokinetic studies reported for ^64^Cu-DOTA-TNYL-RAW in a recent study [49]. In cell culture, TNYL-RAW is degraded by proteases secreted by the cells because peptide loss is delayed in freshly replaced cell culture medium and addition of protease inhibitors blocks its degradation in conditioned medium. Degradation by proteases may also contribute to TNYL-RAW loss *in vivo*, as suggested by the rapid loss of the peptide incubated in mouse serum *ex vivo* [43], [50]. The biodistribution data reported for TNYL-RAW coupled to ^64^Cu-DOTA or polymeric micellar nanoparticles suggest that uptake by the spleen and liver and escretion through the kidneys also contribute to peptide loss from the blood circulation [34], [49].

Despite the low nanomolar EphB4 binding affinity of TNYL-RAW, the very short half-life of the peptide greatly limits its effectiveness, as exemplified by the high concentrations (10–100 µM) needed to inhibit EphB4-ephrin-B2 interaction in cell culture [21], [33], [36], [37]. We used three strategies to improve TNYL-RAW stability in cell culture and circulating half-life: fusion with Fc, complexation with streptavidin and covalent coupling to 40 KDa polymeric PEG molecules. Fusion with Fc has been successfully used to improve the stability and pharmacokinetic properties of numerous proteins and peptides, and a number of drugs approved for human use contain an Fc moiety [39], [40], [51]. Streptavidin has been used in preclinical and human clinical studies as part of tumor-targeting delivery systems, however it cannot be administered multiple times because of its high immunogenicity [41]. Nevertheless, complexation with streptavidin is the most straightforward method to increase the half-life of biotinylated TNYL-RAW and could be used to improve the stability of the peptide for use in cell and organotypic culture systems. It should be noted that although TNYL-RAW-Fc is dimeric and TNYL-RAW-streptavidin is tetrameric, these forms of the peptide remain unable to detectably activate EphB4 and therefore retain the antagonistic function of the monomeric peptide (unpublished data).

Covalent attachment of molecules to PEG, such as 40 KDa branched PEG, is a widely used approach to improve water solubility, shield from proteolytic enzymes, prevent loss through kidney filtration and uptake by the reticuloendothelial system as well as reduce immunogenicity [40], [52], [53], [54], [55], [56]. Therefore, PEGylation greatly prolongs *in vivo* circulating half-lives of peptides and proteins and enhances their therapeutic potential without toxic effects. Indeed, a number of drugs being evaluated in clinical trials or already approved for human use are PEGylated [52], [57], [58].

All three modified forms of TNYL-RAW retain similar high EphB4 binding affinity and inhibitory ability as the non-modified peptide, but have a much increased half-life. The modifications greatly improve the resistance of TNYL-RAW to cell-derived proteases, with essentially no degradation observed for up to two days in PC3 cell-conditioned culture medium. Although the three modifications also all increase TNYL-RAW half-life in the mouse circulation, PEGylation has the most dramatic effect resulting in a circulating half-life of ∼11 hours and a 10 fold higher systemic exposure than the other two modified forms of TNYL-RAW. Furthermore, peptide loss appears to slow down at later times, and ∼20% of the PEGylated TNYL-RAW is still present in the mouse blood 24 hours after intravenous or intraperitoneal administration.

Conjugation of TNYL-RAW to PEG-coated micellar nanoparticles has also been recently shown to increase the half-life of the peptide in the mouse blood circulation to ∼2 hours [49]. Such nanoparticles, filled with an infrared fluorescent dye and labeled with Indium-111, were successfully used to visualize EphB4-expressing prostate xenograft tumors in mice by both optical imaging and single photon emission computed tomography (SPECT). TNYL-RAW coupled to the chelating agent DOTA in complex with Copper-64 (^64^Cu-DOTA-TNYL-RAW) has also been recently used to image EphB4-expressing tumors using small-animal positron emission tomography (PET) [34]. Although the circulating half-life of ^64^Cu-DOTA-TNYL-RAW is much shorter than that of the peptide attached to the nanoparticles [49], it is still sufficient for tumor imaging, perhaps because the N-terminal DOTA chelating group provides some protection from the aminopeptidases present in the blood [50]. These studies support the effectiveness of TNYL-RAW for non-invasive imaging of EphB4-expressing tumors, which could be useful for cancer diagnosis and to monitor the effects of anti-cancer therapies.

PEGylated TNYL-RAW inhibits EphB4 phosphorylation in melanoma cells stimulated with ephrin-B2 even 24 hours after its addition to cell culture medium, whereas the less stable unmodified TNYL-RAW is ineffective after prolonged incubation in cell culture medium. The PEGylated peptide also inhibits tyrosine phosphorylation of EphB4 and ephrin-B2 in co-cultured EMCs and HUVECs after a 15 hour incubation in the culture medium. Furthermore, consistent with increased stability, PEG-TNYL-RAW significantly inhibits endothelial-mural cell assembly into capillary-like tubes at a concentration of less than 1 µM, whereas unmodified TNYL-RAW does not have a significant effect even at a concentration of 20 µM. Interestingly, even at a concentration of 5 µM PEG-TNYL-RAW does not significantly affect capillary-like tube formation in HUVECs or EMCs cultured separately. These findings suggest that while inhibition of EphB4-ephrin-B2 interaction is sufficient to disrupt the assembly of endothelial with perivascular mesenchymal cells under the conditions of our experiments, other Eph receptors may play a more important role in tube formation by HUVECs and EMCs cultured separately [21], [24], [59]. For example, HUVECs express EphB2 [36], another Eph receptor that can stimulate ephrin-B2 reverse signaling and that is not targeted by the TNYL-RAW peptide [33]. In addition, EphA2-ephrin-A1 interaction has been shown to play a critical role in the assembly of HUVECs into tubes [59].

The interaction between EphB4 and ephrin-B2 expressed in endothelial and mural cells also appears to play a critical role in capillary sprouting under the conditions of our experiments, as suggested by the observation that in the absence of exogenously added proangiogenic factors HUVECs and EMCs embedded as spheroids in collagen generate capillary sprouts only when the two cell types are cultured together, an effect that is blocked by the addition of PEG-TNYL-RAW. Interestingly, the sprouts appear to mostly contain EMCs, consistent with reports showing that pericytes in some cases precede endothelial cells in the formation of capillary sprouts [60], [61], [62].

These results imply that the PEGylated form of TNYL-RAW could be used to interfere with tumor angiogenesis and other forms of pathological angiogenesis. The ability of PEG-TNYL-RAW to disrupt the coordinated interplay of endothelial and perivascular mesenchymal cells suggests that this peptide could be used to: (1) enhance the effectiveness of anti-angiogenic cancer treatments targeting vascular endothelial growth factor (VEGF) or VEGF receptor, which preferentially affect smaller blood vessels lacking a perivascular cell component and (2) disrupt the remodeled blood vessels of tumors that have recurred after anti-VEGF treatments, which are typically stabilized by perivascular mesenchymal cells [63], [64], [65], [66]. Interestingly, elevated levels of ephrin-B2 have been reported in the remodeled blood vessels of tumors that recur after anti-angiogenic treatments [65] and the soluble extracellular domain of EphB4 has been shown to disrupt tumor endothelial cell coverage by mural cells *in vivo* [30], [31]. Inhibition of EphB4-ephrin-B2 interaction by PEG-TNYL-RAW could also be useful to inhibit the growth of cancer cells in which EphB4-ephrin-B2 interaction promotes tumorigenesis, such as melanoma cells [9], [10], [11].

Importantly, TNYL-RAW also represents a powerful tool to investigate the role of EphB4-ephrin-B2 interaction in pathological and physiological processes, given its selectivity for EphB4. For instance, TNYL-RAW has been recently used to demonstrate that ephrin-B2 has effects in endothelial cells that are independent of interaction with EphB4 [37] and that EphB4 has effects in breast cancer cells that are independent of interaction with ephrin-B2 [67]. For all these applications, using a form of the peptide with improved stability, such as PEG-TNYL-RAW, will represent an advantage by enabling long-term EphB4 inhibition in cell culture as well as in vivo studies, and the use of substantially lower amounts of the peptide.

## Materials and Methods

### Synthesis of TNYL-RAW and biotinylated TNYL-RAW

TNYL-RAW was synthesized by using solid phase Fmoc [*N*-(9-fluorenyl)methoxycarbonyl] chemistry on a 433A peptide synthesizer (Applied Biosystems, Foster City, CA) with a Tenta Gel S RAM resin, as described previously for other peptides [68]. Biotin was conjugated onto the ε–amino group of a Lys attached at the C terminus of TNYL-RAW through an aminohexanoic acid linker. The crude peptide was purified to ∼95% final purity. Alternatively, TNYL-RAW at ∼90% purity was obtained from Anaspec (Fremont, CA).

### Preparation of TNYL-RAW-Fc, TNYL-RAW-streptavidin and PEG-TNYL-RAW

TNYL-RAW fused with human Fc (TNYL-RAW-Fc) was produced by cloning a cDNA encoding the TNYL-RAW peptide into a pcDNA3-based vector preceded by a signal peptide sequence for secretion into the medium and followed by sequences encoding a GSGSK linker and human Fc [69]. The TNYL-RAW-Fc plasmid was used to transiently transfect human embryonic kidney (HEK) 293 cells, which were expanded in DMEM with 10% fetal bovine serum (FBS) (Hyclone, Logan, UT), and then grown for 4 days in serum-free low IgG Opti-MEM (Life Technologies/Invitrogen). TNYL-RAW-Fc was purified from the cell culture medium using protein A-coupled to sepharose beads (GE Healthcare, UK).

TNYL-RAW in complex with streptavidin (TNYL-RAW-streptavidin) was obtained by incubating biotinylated TNYL-RAW and streptavidin (Pierce Biotechnology, Rockford, IL) at a molar ratio of 4∶1 for 30 min at 4°C in phosphate buffered saline (PBS).

PEGylated TNYL-RAW (PEG-TNYL-RAW) was generated by incubating TNYL-RAW (200–500 µM) with branched 40 KDa PEG-succinimidyl-glutarate molecules (NHS-glutaryl PEG, NOF Corporation, Tokyo, Japan) at a 1∶2 or 1∶3 molar ratio in PBS for 30 min at room temperature followed by 5 hours at 4°C. The PEGylated peptide was then dialyzed for 1.5 days at 4°C against PBS using 5,000 MWCO microdialysis units (Pierce Biotechnology, Rockford, IL) in order to remove peptide that may have remained uncoupled. However, the concentration of PEG-TNYL-RAW after dialysis was estimated assuming that essentially all the peptide was coupled to PEG. A solution containing NHS-glutaryl PEG that underwent all the steps described above but without the addition of TNYL-RAW was used as control in cell-based experiments.

### Preparation of EphB4 AP and ephrin-B2 AP

Alkaline phosphatase (AP) fusion proteins were produced by transiently transfecting the mouse EphB4 AP [70] or mouse ephrin-B2 AP plasmids (GeneHunter, Nashville, TN) in HEK 293T cells and growing the cells in serum-free Opti-MEM medium for 2–4 days. If necessary, the culture medium containing the secreted AP protein was concentrated by centrifugation in Amicon 30,000 MW cutoff filters (Millipore, Inc., Temecula, CA). EphB4 and ephrin-B2 AP concentrations were calculated from alkaline phosphatase activity [71].

### Measurements of TNYL-RAW binding affinity

The EphB4 binding affinity of the different forms of TNYL-RAW was measured in ELISA assays. Different concentrations of biotinylated TNYL-RAW and TNYL-RAW-streptavidin were incubated in TBST buffer (150 mM NaCl, 50 mM Tris-HCl, pH 7.5 with 0.01% Tween 20) in protein A-coated wells (Pierce Biotechnology, Rockford, IL) in which EphB4 Fc (1 µg/ml in TBST; R&D Systems, Minneapolis, MN) had been immobilized. Bound biotinylated TNYL-RAW was detected with horseradish peroxidase (HRP)-conjugated streptavidin (1∶2,000 dilution in TBST, Pierce Biotechnology, Rockford, IL) and bound TNYL-RAW-streptavidin was detected with an anti-streptavidin antibody coupled with HRP (1∶1,000 dilution in TBST, Life Technologies-Invitrogen). Absorbance at 405 nm was measured following incubation with 0.2 mg/ml 2,2′-azino-bis(3-ethylbenzthiazoline-6-sulfonic acid) (ABTS) (Sigma-Aldrich, Steinheim, Germany) in citric acid as a substrate for HRP, and the absorbance in wells without peptide was subtracted as background.

Binding of PEG-TNWL-RAW to EphB4 was measured using a similar ELISA assay, except that Ni-NTA-coated wells (Qiagen, Valencia, CA) were used to immobilize EphB4 Fc (which also contains an hexa-histidine tag). The bound PEG-TNYL-RAW was detected using 2 µg/ml AGP3 anti-PEG antibody (Academia Sinica, Taiwan) in TBST followed by a secondary anti-mouse IgM-HRP antibody (1∶1,000 dilution in TBST, Morphosys, Munich, Germany). Biotinylated PEG-TNYL-RAW could not be detected using streptavidin-HRP, presumably because the bulky PEG interferes with the binding of streptavidin to the C-terminal biotin tag on the peptide. The absorbance from wells without peptide was subtracted as background.

To compare the binding affinities of biotinylated TNYL-RAW and TNYL-RAW Fc, polystyrene high binding capacity plates (Corning, Corning, NY) were coated overnight with either 2 µg/ml streptavidin (Pierce Biotechnology, Rockford, IL) or 10 µg/ml anti-Fc antibody (Jackson laboratory, Sacramento, CA) diluted in borate buffer (0.1 M boric acid, 0.1 M Na borate, pH 8.7) to capture biotinylated TNYL-RAW and TNYL-RAW-Fc, respectively. After blocking with 5 mg/ml bovine serum albumin in PBS, different amounts of EphB4 AP in cell culture medium diluted in TBST were added to the wells for 3 hours. Bound EphB4 AP was detected by adding 1 mg/ml *p*nitrophenylphosphate (pNPP; Pierce Biotechnology, Rockford, IL) in SEAP buffer (105 mM diethanolamine, 0.5 mM MgC1_2_, pH 9.8) as a substrate and measuring the absorbance at 405 nm. The absorbance from wells where no EphB4 AP was added was subtracted as the background. All binding curves were analyzed using non-linear regression and the program Prism (GraphPad Software Inc.).

### Inhibition of EphB4-ephrin-B2 binding

EphB4 Fc (1 µg/ml in TBST) was immobilized in protein A-coated plates and incubated for 3 hours with 0.02 nM ephrin-B2 AP in culture medium diluted in TBST in the presence of different concentrations of TNYL-RAW, TNYL-RAW-Fc, TNYL-RAW-streptavidin and PEG-TNYL-RAW. Bound ephrin-B2 AP was quantified by adding pNPP as the substrate and measuring the absorbance at 405 nm. Alkaline phosphatase activity from wells where human Fc (R&D Systems, Minneapolis, MN) was added instead of EphB4 Fc was subtracted as the background. The binding curves were analyzed using non linear regression and the program Prism (GraphPad Software Inc.).

### Measurement of TNYL-RAW stability in cell culture medium

PC3 prostate cancer cells were grown in RPMI 1640 medium (Mediatech, Inc, Herndon, VA) with 10% fetal bovine serum (FBS) (Hyclone, Logan, UT), penicillin and streptomycin. B16-F10-luc-G5 melanoma cells (Caliper Life Sciences, Hopkinton, MA) were grown in Eagle's MEM with Earle's Balanced Salts (EBSS) (Hyclone, Logan, UT) supplemented with 10% FBS, non essential amino acids (Hyclone, Logan, UT), L-glutamine, sodium pyruvate (Hyclone, Logan, UT), MEM vitamin solution (Life Technologies-Invitrogen), penicillin and streptomycin (Omega Scientific, Tarzana, CA). HUVECs (Cascade Biologics, Portland, OG) were grown in Medium 200 supplemented with low serum growth supplements (Cascade Biologics), 10% FBS, penicillin and streptomycin, and fungizone (Omega Scientific, Tarzana, CA). Rat EMCs were grown in DMEM (Mediatech, Inc, Herndon, VA) supplemented with 10% FBS, penicillin and streptomycin. The different modified forms of TNYL-RAW at a concentration of 25 nM were added to fresh medium or culture medium conditioned by the cells for 1–4 days. The culture medium was collected after different time periods and diluted 1∶10 in ELISA wells. The amount of intact TNYL-RAW peptide remaining was measured by using the ELISA assays described above for measurement of binding affinity. In some experiments, the peptide was incubated at 37°C with PC3 cell conditioned medium collected from the cells in the absence or in the presence of protease inhibitors (10 µg/ml aprotinin, 5 µg/ml pepstatin, 10 µg/ml leupeptin and 0.75 mM or phenylmethylsulfonylfluoride (PMSF)).

### Peptide retention in mouse blood

2.1 nmoles TNYL-RAW-Fc, 1.5 nmoles TNYL-RAW-streptavidin and 6 nmoles PEG-TNYL-RAW, diluted in 150 µl sterile PBS, were administered to C57BL/6 mice by intravenous or intraperitoneal injection. Blood was collected from each mouse from the orbital sinus at two time points after injection and by cardiac puncture under Avertin anesthesia at a third time point. These experiments were carried out in accordance with the recommendations in the Guide for the Care and Use of Laboratory Animals of the National Institutes of Health. The protocol was approved by the Institutional Animal Care and Use Committee of the Sanford-Burnham Medical Research Institute (Animal Assurance Number: A-3053-1). After collection, the blood was clotted fibrinogen, and the serum was stored at −20°C. The different forms of TNYL-RAW in the serum were detected by using the same ELISA assays described above for measurements of binding affinity. The absorbance from wells incubated with mouse serum without TNYL-RAW was subtracted as the background and the concentration of TNYL-RAW in the blood was calculated based on a standard curve generated using serum containing known TNYL-RAW concentrations. The half-life of each peptide was estimated by using non linear regression and the program Prism (GraphPad Software Inc.). The area under the curve (AUC) was expressed as % ID•hours/ml (where ID is the initial dose of peptide injected) and calculated by dividing the AUC value obtained using the program Prism by the amount of injected peptide and multiplying by 100.

### Capillary-like tube formation assays

An EMC clone expressing EGFP was generated by transducing EMCs (a kind gift from H. Eid) with a lentiviral construct followed by clonal selection [46]. To generate capillary-like tubes, HUVECs (2.8×10^4^), EMCs (7.5×10^4^) or a mixture of the two cell types (2.8×10^4^ HUVECs and 1.4×10^4^ EMCs) were grown for 5 to 20 hours in 24-well tissue culture plates (Corning, Corning, NY) precoated with 80 µl Matrigel (BD Bioscience, San Jose, CA) in Medium 200 or DMEM complete culture medium containing PEG-TNYL-RAW or PEG control. In some experiments, mixed cultures were treated with TNYL-RAW or DMSO control. The cells were then fixed for 15 min in 4% formaldehyde in PBS and photographed under a fluorescence microscope. Tube length was quantified using ImageJ software.

### Immunofluorescence

HUVECs, EMCs or a 2∶1 mixtures of the two cell types were cultured for 15 hours on untreated or Matrigel-coated glass coverslips and stained with an anti-smooth muscle actin antibody (Sigma-Aldrich, Steinheim, Germany), followed by a secondary anti-mouse antibody conjugated with Alexa Fluor 568 (Life Technologies/Invitrogen). To assess the effect of PEG-TNYL-RAW on ephrin-B2 phosphorylation, HUVECs and EMCs were plated at a 1∶1 ratio on glass coverslips in complete Medium 200 in the presence of PEG-TNYL-RAW or PEG control. After 15 hours, the cells were stimulated with 1.5 µg/ml EphB4 Fc or Fc control, which had been preclustered with a 3 fold excess of an anti-Fc antibody for 20 min in the continued presence of the peptide. The cells were then fixed for 15 min in 4% formaldehyde in PBS, permeabilized for 3 min in 0.5% Triton X-100 in TBS, and stained with anti phospho-ephrin-B (Tyr324/329) antibody (Cell Signalling, Danvers, MA) followed by a secondary anti-rabbit antibody conjugated with Alexa Fluor 568. Nuclei were labeled with 4′,6-diamidino-2-phenylindole (DAPI) and the cells were photographed under a fluorescence microscope. To quantify the amount of phosphorylated ephrin-B2, total fluorescence intensity from 6 representative micrographs was measured using Metamorph software (Molecular Devices) and normalized to the number of cells in each micrograph.

### Capillary sprouting assay

Spheroids were generated by seeding 750 HUVECs transduced with a lentiviral vector encoding mCherry (GeneCopoeia, Rockville, MD), EMCs expressing EGFP or a 1∶1 mixture of the two cell types in 20 µl of complete Medium 200 in non-adherent round bottom 96 well plates (Corning, Corning, NY). The plates were centrifuged at 160 g for 25 sec and placed in a 5% CO_2_ cell culture incubator at 37°C. After 24 hours, PEG-TNYL-RAW or control PEG were added in 5 µl FBS and the spheroids were embedded in collagen by adding to each well 20 µl of 2 mg/ml neutralized rat tail collagen according to a published protocol [72]. After allowing the collagen to polymerize for 1 hour at 37°C, complete medium without added angiogenic factors and containing PEG-TNYL-RAW or PEG was added on top of the collagen. The embedded spheroids were kept for 2 days in a 5% CO_2_ cell culture incubator at 37°C and photographed under a fluorescence microscope. The number and total length of capillary sprouts originating from each spheroid was quantified using ImageJ software.

### Immunoprecipitation and immunoblotting

B16-F10-luc-G5 melanoma cells, which endogenously express EphB4, were serum starved for 1–3 hours in Eagle's MEM/EBSS containing 0.5% FBS and incubated for 15 min or 24 hours with different concentrations of PEG-TNYL-RAW. The cells were then stimulated for 20 min with 1.5 µg/ml ephrin-B2 Fc, which had been preclustered with a 3 fold excess of an anti-Fc antibody, in the continued presence of the peptide. HUVECs, EMCs or a 1∶1 mixture of the two cell types were plated for 20 hours in complete medium in the presence of PEG or PEG-TNYL-RAW. Medium 200 was used for HUVECs and HUVECs and EMCs co-cultures, DMEM was used for EMCs. The cells were lysed in modified RIPA buffer (1% Triton X-100, 1% Na deoxycholate; 0.1% SDS; 20 mM Tris; 150 mM NaCl; 1 mM EDTA) containing 10 µM NaF, 1 µM sodium pervanadate and protease inhibitors. Protein concentrations were measured using the BCA protein assay kit (Pierce Biotechnology, Rockford, IL). For immunoprecipitations, cell lysates were incubated with 20 µg of an anti-EphB4 antibody made to a GST fusion protein of the EphB4 SAM domain and carboxy-terminal tail [15] immobilized on GammaBind Sepharose beads (GE Healthcare Life Sciences). Immunoprecipitates and lysates were probed by immunoblotting with anti-phosphotyrosine antibody (Millipore, Inc., Temecula, CA), pan-ephrin-B antibody (Life Technologies/Invitrogen), anti-ß-actin antibody (Sigma-Aldrich, Steinheim, Germany), and anti-EphB4 antibody.

## Supporting Information

Figure S1 **The TNYL-RAW peptide is rapidly lost in cell culture medium from different cell types.** Biotinylated TNYL-RAW was added to cell culture medium collected from B16 melanoma cells, HUVECs and EMCs after overnight culture. Functional (EphB4- and streptavidin-binding) peptide remaining at the indicated times was captured in ELISA plates coated with EphB4 Fc and detected with streptavidin-HRP.
(TIF)

Figure S2 **EMCs express smooth muscle actin.** EMCs expressing EGFP were cultured on coverslips (top panels) or coverslips coated with Matrigel (bottom panels) and stained for α-smooth muscle actin and with DAPI to label nuclei. HUVECs were also stained as a negative control. Scale bars = 100 µM.
(TIF)
