# Supplementary figures and images for: PEGylation Potentiates the Effectiveness of an Antagonistic Peptide That Targets the EphB4 Receptor with Nanomolar Affinity
Source: PLoS One. 2011 Dec 14;6(12):e28611. doi: 10.1371/journal.pone.0028611 (PMC3237458; doi:10.1371/journal.pone.0028611)

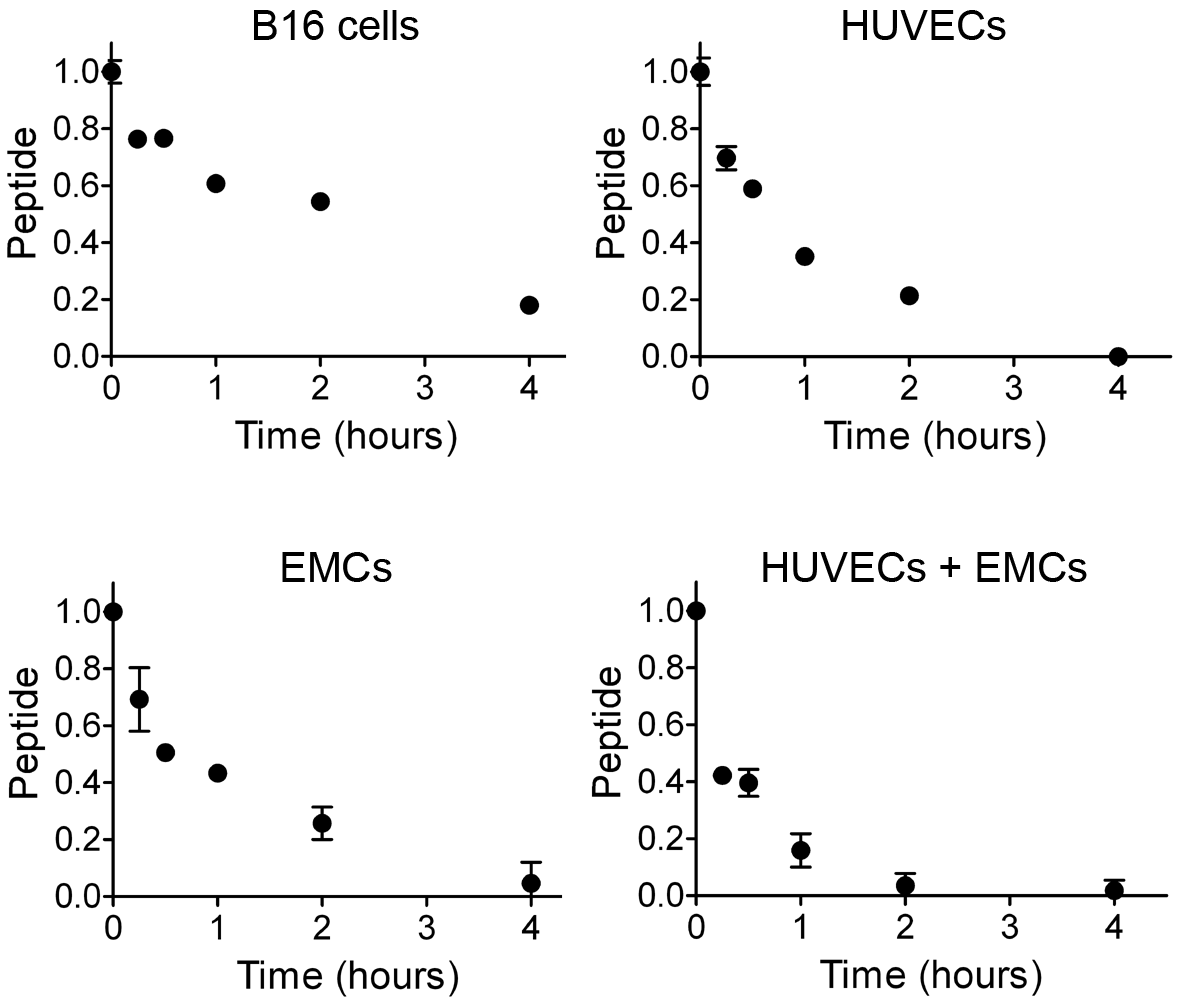

Supplement: Figure S1 — The TNYL-RAW peptide is rapidly lost in cell culture medium from different cell types. Biotinylated TNYL-RAW was added to cell culture medium collected from B16 melanoma cells, HUVECs and EMCs after overnight culture. Functional (EphB4- and streptavidin-binding) peptide remaining at the indicated times was captured in ELISA plates coated with EphB4 Fc and detected with streptavidin-HRP. (TIF) [file pone.0028611.s001.tif]

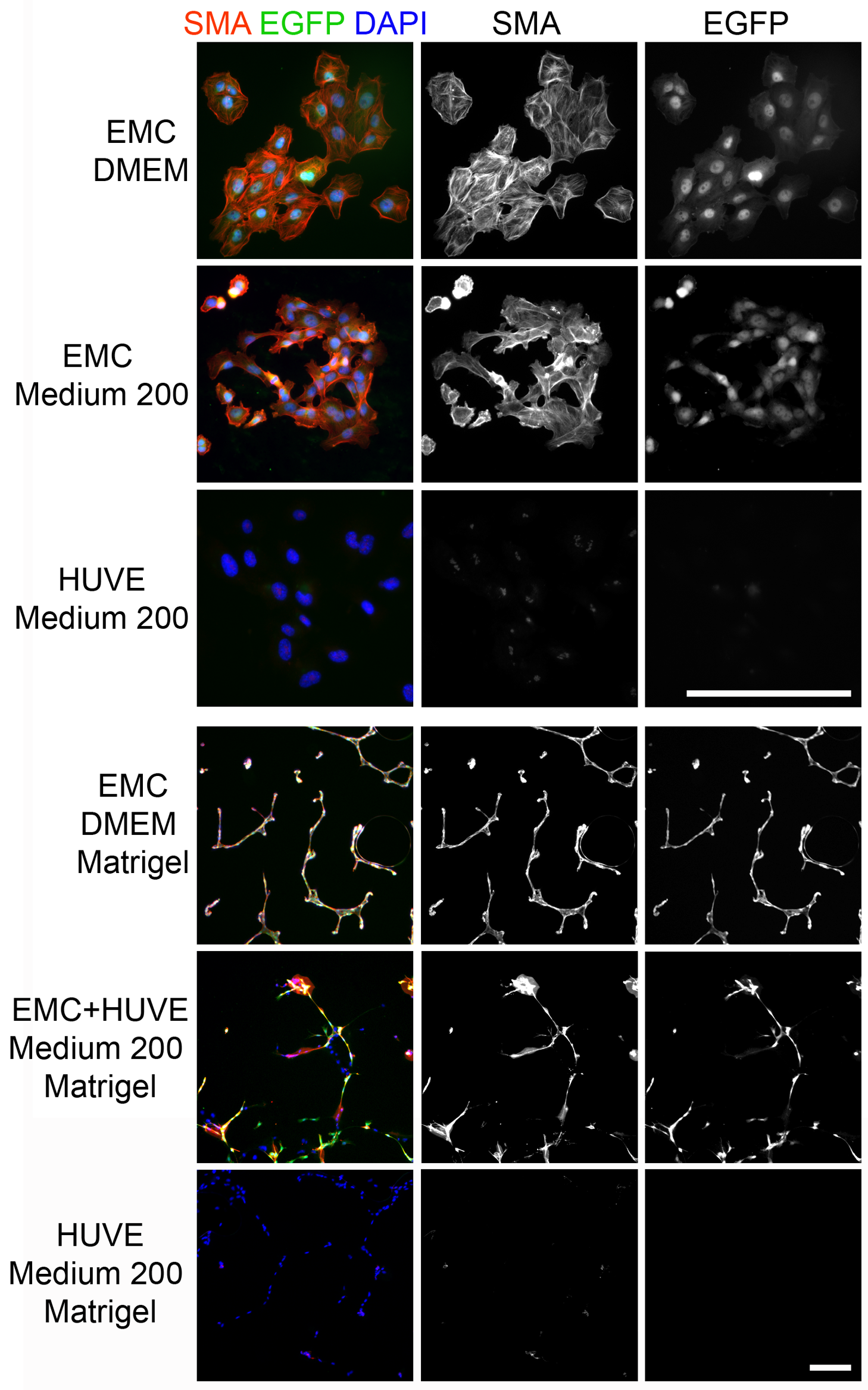

Supplement: Figure S2 — EMCs express smooth muscle actin. EMCs expressing EGFP were cultured on coverslips (top panels) or coverslips coated with Matrigel (bottom panels) and stained for α-smooth muscle actin and with DAPI to label nuclei. HUVECs were also stained as a negative control. Scale bars = 100 µM. (TIF) [file pone.0028611.s002.tif]
